# Assembly complexity of prokaryotic genomes using short reads

**DOI:** 10.1186/1471-2105-11-21

**Published:** 2010-01-12

**Authors:** Carl Kingsford, Michael C Schatz, Mihai Pop

**Affiliations:** 1 Department of Computer Science and Center for Bioinformatics and Computational Biology, Institute for Advanced Computer Studies, University of Maryland, College Park, MD, USA

## Abstract

**Background:**

De Bruijn graphs are a theoretical framework underlying several modern genome assembly programs, especially those that deal with very short reads. We describe an application of de Bruijn graphs to analyze the global repeat structure of prokaryotic genomes.

**Results:**

We provide the first survey of the repeat structure of a large number of genomes. The analysis gives an upper-bound on the performance of genome assemblers for *de novo *reconstruction of genomes across a wide range of read lengths. Further, we demonstrate that the majority of genes in prokaryotic genomes can be reconstructed uniquely using very short reads even if the genomes themselves cannot. The non-reconstructible genes are overwhelmingly related to mobile elements (transposons, IS elements, and prophages).

**Conclusions:**

Our results improve upon previous studies on the feasibility of assembly with short reads and provide a comprehensive benchmark against which to compare the performance of the short-read assemblers currently being developed.

## Background

Recently, new technologies developed by 454 Life Sciences [1], Illumina [2], ABI/SOLiD [3], Helicos [4], and others can sequence large quantities of DNA in only hours. Instead of approximately 1000 nucleotide reads common with traditional Sanger sequencing, these technologies generate reads of only about 25-400 consecutive nucleotides. Further, with traditional sequencing techniques, most of the ~ 1000 nt reads come in pairs, separated by an approximately known distance spanning thousands or tens of thousands of nucleotides. These pairs provide long-range information to help order the reads, but such long-range pairing is not yet widely available or is considerably more expensive. The next generation sequencing technologies with paired-end protocols are generally limited to much more closely spaced pairs, typically separated by at most a few hundred or a few thousand nucleotides. Nevertheless, these limitations are mitigated by much higher throughput (approximately 4 hours per 500 × 10^6 ^nucleotides of DNA for 454-based sequencing, for example) and the resulting increased redundancy that comes from the ability to cheaply sample the same genome many times over. However, shorter read lengths lead to a much more computationally challenging assembly problem because they provide less information to determine the location and structure of repetitive subsequences. It is therefore important to understand the limits and promise of these technologies.

In this paper, we study an idealized instance of the short-read assembly problem and attempt to estimate the ability of these technologies to reconstruct genomes. In particular, for a given read length *k*, we assume that we are given all length-*k *substrings of a genome *g *as well as the number of times they occur. This is an idealization in many respects. First, in practice not all length-*k *substrings will be obtained. Second, many of the substrings returned by the sequencing technology will contain errors (indels or substitutions). However, the repetitive sampling possible with the faster and less expensive sequencing reduces these two problems somewhat. Furthermore, the topic of error correction on short-read sequences is itself an active area of research (see, e.g. [5]). Third, sequencing technologies return substrings of both *g *and the reverse complement of *g*. Here, we assume instead that the reads are all oriented in the forward direction. Lastly, sequencing technologies do not directly provide the number of occurrences of each length-*k *substrings. Instead, the number of occurrences must be estimated from statistics computed on the population of redundantly sampled length-*k *substrings. For example, sequences observed twice as often as expected probably occur twice in the genome.

Despite these simplifying assumptions, it is important to conduct an analysis of the fundamental challenge of assembly -- the repeat structure of the genome -- free of the biases or limitations of any particular sequencing technology. These technologies are advancing at a rapid rate, and while the sequencing error rate or read length may improve from one model of sequencer to the next, the repeat structure of a genome is fixed. Our analysis shows the fundamental limits of assembly based on just one unavoidable parameter, the length of the reads, which in turn determines the length of confounding repeats. Thus we can give an upper bound on how well an assembler could possibly reconstruct a genome given reads of a certain length. In practice, an assembly of real data may fail to reach this limit by a considerable margin, but we can be confident that no assembler could surpass this limit without risking misassemblies. Furthermore, our analysis also gives an upper bound on assembly with paired-end sequences, which we can conservatively model as extremely long reads, because reads of length *X *have strictly more information than paired-end sequences with insert size *X*.

Similar string reconstruction problems have been considered in the past [6,7]. Rubinov and Gelfand [8] considered the problem of reconstructing a string given the set of *k*-long substrings and a list of all pairs of *k*-long subsequences (*i, j*) such that *i *precedes *j *at some distance in the string. Because they have complete, long-range precedence data, the problem is different than that which we consider here. Others [7] have considered the problem of reconstructing a string from its length-*k *substrings taking their multiplicities {*c*_*i*_} as unknown (rather than as input as we do here). The problem of finding the shortest superstring containing a set of strings has also been extensively studied (see, e.g. [9]). Of course, work in traditional sequence assembly has produced many algorithms and heuristics (e.g. [10-12]) for solving similar problems in practice. Newer assemblers are also being developed to handle short-read data (e.g. [5,13-18]).

In this paper, we explore the repeat structure of a large collection of prokaryotic genomes at different levels of resolution. We define a measure of the complexity of a genome in terms of the number of possible different reconstructions with the same repeat structure for repeats longer than a given threshold (read length). Our measure complements previously proposed complexity measures that estimate the "repetitiveness" of a genome's sequence at a local scale (linguistic complexity [19]) or global scale (index of repetitiveness [20]) and focuses specifically on the question of how repeats affect the ability to reconstruct a genome from reads of a given length. For example, our method would distinguish between the difficulty of assembling 3 repeats each in 2 copies (8 different genome reconstructions) from the difficulty of assembling one repeat with 6 copies (720 different reconstructions) while a simple repeat counting method might not. Furthermore, global repeat analysis might fail to take into account the relative positioning of the repeats. We reveal that most of the repeat complexity of the majority of organisms is caused by intergenic repeats or mobile genetic elements. We conclude from this analysis that most genes in a prokaryotic organism can, in principle, be reconstructed from reads as short as 25 nt. This is in contrast with the difficulty even a small number of repeats poses on the reconstruction of the entire genome.

This study represents a first comprehensive empirical investigation of the limits of the information that can be extracted from short reads encoded within de Bruijn graphs. In this context, a de Bruijn graph for length-*k *has a node for each (*k *- 1)-substring in the read set and an edge connecting two nodes if their (*k *- 1)-mers are consecutive in some read. The de Bruijn graph has become a popular framework for genome assembly of short reads, since the graph structure captures the sequence and repeat composition of the genome, without requiring an expensive explicit overlap computation between reads. Here, we quantify how easily prokaryotic genomes can be reconstructed from short-read data by using several previously known combinatorial results to compute the number of strings consistent with length-*k *substrings extracted from 375 organisms. Using these exact combinatorial results, we improve upon previous estimates of assembly complexity [15,21] that relied primarily on the distribution of intra-repeat distances. We also derive another estimate for the complexity of the assembly problem by computing an upper bound on the N50 contig size that is achievable, even with mate-pair information. (The N50 contig size is a widely used statistic for evaluating the quality of a proposed assembly.) These more accurate estimates of the complexity of the reconstruction problem permit a more realistic assessment of the challenges of the assembly problem and provide a benchmark against which new assembly methods can be evaluated.

## Methods

### Constructing the de Bruijn graph

The de Bruijn graph derived from length-*k *reads of a genome *g *contains a node for each (*k *- 1)-mer present in the genome, and a directed edge *u *→ *v *for every instance where the (*k *- 1)-mer represented by *v *occurs immediately after the (*k *- 1)-mer for *u*. In other words, there is an edge if *u *occurs at position *i *and *v *occurs starting at position *i *+ 1. These edges can be obtained by considering every *k*-mer present in the genome and connecting the node for its (*k *- 1)-prefix to the node for its (*k *- 1)-suffix. Crucially, the de Bruijn graph can be a multigraph, with parallel edges between nodes. Self-loops are also permitted. Note that this definition of a de Bruijn graph differs from the traditional definition described in the mathematical literature in the 1940s [22] that requires the graph to contain all length-*k *strings that can be formed from an alphabet (rather than just those strings present in the genome). The more general formulation of the de Bruijn graph used in this paper, and the closely related string graph, are commonly used in the sequence assembly literature [23-26] under the same name, and we follow the same convention. Throughout this paper, we use *s, t, u, v *and *w *to denote both nodes within the de Bruijn graph and the DNA sequences they represent. The de Bruijn graph encodes all the information available from the input of the list of all *k*-mers of *g*. Because an edge *u *→ *v *only occurs if *v *follows *u *somewhere in the genome *g*, we must traverse every edge to reconstruct the genome, and thus the correct genome sequence corresponds to an Eulerian path through the de Brujin graph [27].

We classify the nodes of a de Bruijn graph as *decision *and *non-decision *nodes. Decision nodes--those with more than one predecessor or more than one successor--are the primary complication in reconstructing the correct genome sequence from a de Brujin graph as they introduce ambiguity in possible graph traversals. Non-decision nodes (with at most one successor and at most one predecessor) correspond to sections of the graph that can be unambiguously reconstructed.

### Simplification of the de Bruijn graphs

**Figure 1:**
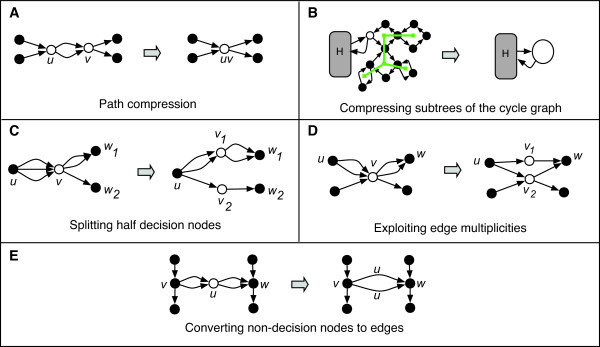
**Graph Transformations**. Each transformation modifies the genome graph such that the set of sequences consistent with the graph is unchanged. The nodes duplicated or removed by the simplification are shown as hollow circles. (a) Standard path compression collapses adjacent nodes *u *and *v *if *v *must follow *u *and *u *must precede *v*. (b) Portions of the cycle graph that are trees (shown as lines connecting square nodes) represent sections of the genome graph with a single solution. These can be collapsed into a single node connected to the rest of the sequence graph *H*. (c) Forward and backward half-decision nodes (those with either a single predecessor or a single successor) can be split into several nodes, which can usually be eliminated with path compression. (d) We can infer a path between a predecessor and successor if reasoning akin to the pigeonhole principle implies that at some point that predecessor must immediately precede that successor. (e) Some non-decision nodes *u *cannot be eliminated via path compression because both their predecessor and successors are decision nodes. In these cases, we can eliminate the non-decision node, and several edges, by replacing *u *with edges labeled with the sequence represented by *u*.

We applied the following graph transformations to the idealized de Bruijn graph constructed from the genome sequences. Each transformation preserves the entire set of paths consistent with the graph. See Figure 1 for illustrations of the various transformations. Successively applied, these transformations simplify the graph towards the smallest equivalent graph possible -- the most parsimonious graph structure that encodes the full information contained in the set of *k*-mers.

We use the term *path *to be an ordered sequence of edges. If the path starts and ends at the same node, it is a *circuit*. A *trail *is a path that is not a circuit. If the nodes *s *and *t *with unequal in- and out-degrees exist then the de Bruijn graph admits an Eulerian trail starting at *s *and ending at *t*, otherwise it contains only Eulerian circuits. Either is possible for graphs obtained from genomic sequence data: a circular chromosome necessarily yields a graph with circuits, while a linear genome may or may not, depending on whether the 3'-most *k *- 1 nucleotides equal the 5'-most.

#### Compressing paths

If *u *→ *v *is an edge, we say *u *is a *predecessor *of *v *and *v *is a *successor *of *u*. If a node *u *has a successor *v*, and if *u *is the only predecessor of *v*, then nodes *u *and *v *can be merged into a single node that has the predecessors of *u *and the successors of *v *(Figure 1a), similar to previous approaches [14-17]. This transformation, called *path compression*, can be made even if there are multiple, parallel edges from *u *to *v*. Prior to path compression, there is a node for each (*k *- 1)-mer that appears in the genome, and the number of edges equals the genome length. Following path compression, the graphs dramatically decrease in size as non-branching chains are replaced by single nodes.

Path compression is a standard, natural technique, but additional simplifications are also possible for reducing the size of the genome graph even further. Decision nodes--those with more than one predecessor or more than one successor--are the complication in extracting the correct genome sequence from a genome graph. We distinguish three types of decision nodes: *forward *decision nodes have more than one successor, but a single predecessor; *backward *decision nodes have more than one predecessor, but a single successor; and *full *decision nodes have both more than one predecessor and more than one successor. We refer to forward and backward decision nodes as *half *decisions nodes.

#### Compressing tree-like regions

Any Eulerian graph *G *can be decomposed into simple, edge-disjoint cycles. (A simple cycle is one that uses each node at most once.) From this decomposition, a *cycle graph ***cycle**(*G*) can be constructed with a vertex for each simple cycle in *G *and an edge connecting cycles if they share a node in the Eulerian graph. If **cycle**(*G*) is a tree, then *G *only has a single Eulerian tour [28]. More generally, if an induced subgraph of **cycle**(*G*) is a tree, then the corresponding region has a unique traversal in any Eulerian tour. Thus, these tree-like regions can be collapsed into a single node labeled by the sequence constructed from that unique tour (Figure 1b).

Rather than explicitly constructing the cycle graph, in practice it is more efficient to recursively collapse the tree-like regions, starting with the leaves. Leaves can be identified by pairs of nodes *u*, *v *for which *u *is both the only predecessor and only successor of *v*, and *u *has only a single in-edge and a single out-edge that are not adjacent to *v*. In this case, the sequence of *v *can be appended to *u*, and *v *can be eliminated. A node *u *that has one or more self-loops and has only one in- and one out-edge that are not self-loops is also a leaf, and its self-loops can be collapsed into *u*. After collapsing a leaf, we perform any newly possible path compressions near the collapsed node.

#### Splitting half decision nodes

The graph can be modified by splitting forward and backward decision nodes into several new nodes without changing the strings represented by the graph (Figure 1c). If *v *is a forward decision node with predecessor *u *and successors *w*_1_, ..., *w*_*m *_(*m *≥ 2) then node *v *can be replaced by *m *new nodes *v*_1_, ..., *v*_*m*_. Edges *v*_*i *_→ *w*_*i *_are added with multiplicity equal to the multiplicity of the edge *v *→ *w*_*i*_. Backward decision nodes can similarly be split. Splitting a forward decision node in this manner may cause its predecessor to become a forward decision node, which can subsequently be split. In this way we can "unzip" a sequence of half decision nodes, thereby shifting the decision point in the direction of the unique predecessor/successor. While this transformation increases the number of nodes, the new nodes *v*_*i *_can often be eliminated by an application of path compression.

#### Exploiting edge multiplicities

If, at each decision node, each incoming edge could be correctly paired with an outgoing edge, there would be no difficulty in reconstructing the correct genome. Typically, without additional information, it is not possible to make any such pairings. In some cases, however, the edge multiplicities can be used to identify a predecessor of a node that must be matched with a successor (Figure 1d). In particular, let *u *→ *v *→ *w *be three nodes in a path such that the edge *u *→ *v *has the highest multiplicity among edges entering *v *and *v *→ *w *has the highest multiplicity among edges leaving *v*. Let *c*_*u *_and *c*_*w *_be the multiplicities of the edges *u *→ *v *and *v *→ *w*, respectively. If *u *≠ *w*, we can infer that the path *u *→ *v *→ *w *must be part of any Eulerian tour if *c*_*u*_> *d*^+^(*v*) -*c*_*w*_, where *d*^+^(*v*) is the number of edges leaving *v*. In other words, all the incoming edges coming from *u *cannot be matched without using at least one outgoing edge adjacent to *w *(using reasoning similar to the pigeonhole principle). This reasoning can be used to reconstruct longer subsequences of DNA, but because it generally increased the size of the final graphs due to interactions with the other simplification techniques described here, we do not consider this type of simplification further in this paper.

#### Converting non-decision nodes to edges

Repeated application of these transformations typically leads to many three-node paths *u *→ *v *→ *w *where *u *is the only predecessor of *v *and *w *is the only successor of *v *but, because there are other edges incident to *u *and *w*, the path cannot be collapsed. We can, however, replace node *v *with an edge *u *→ *w *labeled with the sequence of *v *(Figure 1e). Following path compression and this transformation, the graph contains either just a single node or only *decision nodes *that have both more than one predecessor and more than one successor.

#### Maximal compression

The simplification procedures aim to: (i) coalesce multiple adjacent non-decision nodes into single nodes or edges; and (ii) resolve some of the apparent ambiguity represented by the decision nodes. In order to estimate the smallest graph that is equivalent to the input de Bruijn graph, we apply each of these transformations in turn. Because several of the transformations can lead to opportunities for new applications of other transformations, the order in which we apply them is important. We start by performing path compression. Then, all tree-like regions of the graph are collapsed, followed by another round of path compression. We then split all backward decision nodes, then all forward decision nodes, and perform the path compressions that have been newly made possible. Finally, we convert non-decision nodes into edges. These techniques reduce, on average, the number of edges in the de Bruijn graphs by a further 65% compared with performing only path compression.

### Counting words consistent with de Bruijn graphs

In 1975, J.P. Hutchinson and H.S. Wilf [29,30] gave an expression that can be used to compute the number of unique words consistent with a de Bruijn graph. The following theorem, from [30], gives an expression for the number of possible reconstructions that are consistent with the de Bruijn graph. Let *d*^-^(*u*) and *d*^+^(*u*) be, respectively, the in- and out-degrees of vertex *u *(where the graph will be clear in context). Because the de Bruijn graph is Eulerian, *d*^+^(*u*) = *d*^-^(*u*) for all *u *with the possible exception of two nodes *s *and *t *for which *d*^-^(*s*) = *d*^+^(*s*) - 1 and *d*^+^(*t*) = *d*^-^(*t*) - 1.

**Theorem 1 (Adapted from **[30]) *Let A *= (*a*_*uv*_) *be the adjacency matrix for an n-vertex de Bruijn graph G, with both a*_*uv *_> 1 *and self-loops allowed. If d*^+^(*v*) = *d*^-^(*v*) *for all v, then choose a vertex t arbitrarily, otherwise pick the unique t such that d*^+^(*t*) = *d*^-^(*t*) - 1. *Finally, let r*_*u *_= *d*^+^(*u*) + 1 *if u *= *t or r*_*u *_= *d*^+^(*u*) *otherwise. Then the number of words consistent with G that can be spelled ending with node t is given by*(1)

*where L is the n × n matrix with r*_*u*_-*a*_*uu*_* along the diagonal and -a*_*uv*_* in entry *(*u, v*).

The values *r*_*u *_in Theorem 1 are the number of times that the sequence represented by *u *must appear in the output word, given that path for the word ends at node *t *and thus that the output word ends with the sequence represented by *t*. When traversing the graph, we output *u *when we exit node *u*, except for the last node on a trail or circuit which must also be output when it is entered for the last time so that the *k*-mer associated with the last traversed edge is output. See [29,30] for a proof of the above theorem. Briefly, it is proved by an extension of the "BEST" theorem [31-33] for counting the number of Eulerian circuits modified to correct for multiple edges and for the fact that we allow Eulerian trails in addition to only circuits. For example, the sequence *q *= "sababababab" yields a three node de Bruijn graph when *k *= 5. For this graph, the number of tours is 3!3! = 36 but they all yield the same sequence. By increasing the length of *q *in this example, we can increase the number of Eulerian circuits arbitrarily, while maintaining the property that all circuits reconstruct a single word. (*G, t*) is computable in polynomial time.

To correctly apply Theorem 1 to the short-read assembly problem, we have to consider the cases of linear and circular genomes separately. In the typical circular case, the graph *D*_*k*_(*g*) will contain Eulerian circuits, and any node can be chosen as the final node *t*. Theorem 1 will count the number of distinct linear words *q *ending at *t*. If *t *occurs more than once in the genome but there is some unique sequence of DNA in the genome, then cycling *q *to end at each instance of *t *yields a different word counted by (*D*_*k*_(*g*), *t*), each of which is equivalent to the same cyclic ordering of the nodes. In this case, because there are *d*^+^(*t*) occurrences of *t*, the number of cyclically equivalent words starting at *t *is (*G*_*k*_(*g*), *t*)/*d*^+^(*t*).

We say a de Bruijn graph of a circular genome is *periodic *if it can be traversed with an Eulerian path of the form (*tw*_1_*tw*_2_*t*... *tw*_*m*_)^*c*^*t *for *c *> 1 and some choice of (possibly empty) paths *w*_1_, ..., *w*_*m *_that do not contain *t*. (The exponentiation notation indicates *c *repetitions of the parenthesized path.) In the very special case of periodic, circular genomes the number of solution words does not equal (*D*_*k*_(*g*), *t*)/*d*^+^(*t*) for each *t *because many words may be cyclic permutations of each other. We do not encounter this special case in the chromosomes studied here. In particular, if there is any unique sequence of DNA of length ≥ *k *- 1 then the graph will not be periodic.

In the less common case of linear genomes, if the graph admits an Eulerian trail, there is a single choice of which node to chose as *t*. If a linear genome yields an Eulerian graph with Eulerian circuits (because its *k *- 1 suffix equals its *k *- 1 prefix), then any node can be used as the final node *t*, but the linearization of the Eulerian circuit may produce a chromosome with the wrong start position, a minor misassembly.

### Extracting idealized contigs and computing the N50 size

Every edge in the genome graph corresponds to a sequence of DNA in the chromosome. Initially, these edges correspond to *k*-mer reads, but after path compression and other simplifications discussed above, these edges can represent long, unambiguous stretches of the chromosome bounded on either end by ambiguities. Hence, it is reasonable to use the set of remaining edges to estimate a set of contigs that can be extracted from a genome graph. We create one contig for each edge. If non-decision nodes are collapsed into edges as described above, some edges will be labeled with DNA sequences. If edge *u *→ *v *has been labeled, the contig associated with an edge *u *→ *v *will contain the sequence represented by node *u *concatenated with the sequence assigned to edge *u *→ *v*, otherwise the contig will contain only the sequence represented by node *u*. If *u *→ *v *is labeled, then the length of the contig is taken to be **length**(*u*) + **length**(*u, v*) - 2(*k *- 2). If *u *→ *v *is unlabeled, the length of the contig is taken to be **length**(*u*) - (*k *- 2). Because the sequences represented by adjacent nodes and edges overlap by *k *- 2 nucleotides, the terms (*k *-2) and 2(*k *-2) ensure that overlapping bases are counted only once. Hence, the sum of the lengths of the contigs equals the chromosome length. The *N50 size *is the length *m *of the largest contig such that at least 50% of the genome is covered by contigs of size ≥ *m*; it is a commonly used measure of the connectedness of the assembly. Computing the N50 size using the idealized contigs defined above gives an estimate for the achievable N50 size. This estimate will probably be much larger than what can actually be obtained from real, noisy data, and thus it provides a reasonable upper bound on that size in practice.

### Counting reconstructible genes

For many purposes, it is sufficient to reconstruct only the coding regions of the genes of a species. We consider a gene to be reconstructible if the sequence encoding it can be unambiguously inferred from the de Bruijn graph to be present in the genome. For example, if a gene contains a repeat, it is less likely to be reconstructible because the repeat will introduce several possible sequences downstream of the start codon.

Formally, to test whether a gene is reconstructible, we first find the node *s *that represents the region of the chromosome containing the start codon of the gene. We then find all paths that start at *s *and continue until they pass through first a backward decision and then a forward decision. For this purpose, a full decision node counts as simultaneously a backward and forward decision. If the region of the gene is wholly contained within a region represented by such a path, then we say the gene is reconstructible. This is a local definition of reconstructibility, requiring only a neighborhood around the node containing the start codon *s *to be considered. For the analysis presented here, we test each of the genes for reconstructibility on a graph in which the tree-like regions have been collapsed and path compression has been performed (see above).

Forward decisions are not by themselves sufficient to confuse the reconstruction: because every edge must be taken, we can exhaustively follow each path. Nodes with more than one predecessor (backward decisions), however, "pollute" the walk and once a subsequent forward decision is encountered it is not possible to locally determine which branch should be taken. It is possible that a gene may not be considered reconstructible when walking from its start codon to its stop codon but would be reconstructible if the definition were changed to consider walking from its stop codon to its start codon. In practice, we expect few genes are reconstructible by walking forward but not reconstructible by walking backward, hence we restrict ourselves to the definition of reconstructibility above.

## Results and Discussion

### Sequences and annotations

Sequence and annotation data for 384 completely sequenced bacterial and archaeal genomes were downloaded from GenBank. These genomes comprise 668 chromosomes and plasmids. Our study was focused on the main chromosomes of these organisms, so molecules whose FASTA header line included the word "plasmid" were excluded. This left 419 molecules, of which 11 were linear DNA. For simplicity, these 11 linear molecules were excluded from subsequent analysis, leaving 408 molecules representing the chromosomal DNA of 375 organisms. The annotation in the .ptt file accompanying the sequence was used when evaluating how many protein-coding genes could be uniquely reconstructed.

### Simplification of de Bruijn graphs from prokaryotic genomes

We constructed de Bruijn graphs representing the main chromosomes of 375 organisms (408 chromosomes in total) from all substrings (reads) of length 25, 35, 50, 100, 250, and 1000, as described in the Methods section. This resulted in representations of the chromosomes at 7 different resolutions. This process simulates perfect genome coverage with error-free reads. Every path through this graph represents a possible reconstruction of the genome that is consistent with the read information. The initial de Bruijn graphs, obtained from the set of length-*k *substrings, are typically very large and difficult to analyze computationally. Therefore, we first simplify the graph as much as possible through a series of graph transformations, as described in the Methods section above.

The simplification techniques are extremely effective in reducing the size of the genome graphs without changing the information they encode. After performing an initial round of path compression, compressing tree-like regions of the graph that have a unique traversal further reduced the number of edges in each genome graph by between 8% (25 nt reads) and 18% (1000 nt reads) on average. These averages are somewhat skewed by a number of already small graphs that experienced a near-100% reduction in number of edges. The median reduction in number of edges ranged from 2.5% (1000 nt reads) to 9.5% (100 nt reads) and is shown in Table 1 for various read lengths. Splitting half decision nodes (nodes that have a single successor but multiple predecessors, or a single predecessor but multiple successors) into several parallel paths and compressing any newly compressible paths provides a large further decrease in the size of the graph compared with the number of edges following the compression of tree-like regions. This splitting and compression gives further median reductions of between 20 and 26 percent. The reduction factor does not strongly depend on the read length. Table 1 gives the median reduction in the number of edges achieved by this node splitting. Finally, some non-decision nodes with only a single predecessor and single successor will remain. Converting these nodes to labeled edges causes the number of edges to be reduced by approximately another 50% beyond the already applied reductions. The combined effect of all simplifications was to reduce the number of edges by a median of between 65 and 70 percent (Table 1), creating much smaller and more manageable graphs, from which longer contiguous sequences can be extracted.

**Table 1:** Reduction in graph sizes.

*k*	Collapsing Trees (%)	Splitting half-decisions	Total reduction (%)
25	4.56	25.98	64.78
35	6.34	23.11	65.17
50	8.12	21.34	65.87
100	9.54	20.18	67.01
250	6.50	24.21	68.21
500	4.76	25.93	70.31
1000	2.50	25.00	70.00

Column 2 gives the median percent reduction in number of edges by compressing tree-like regions compared with performing only path compression. Column 3 gives the median further reduction in number of edges achieved by splitting half-decision nodes, for various read lengths *k*. Column 4 gives the median percent reduction in number of edges (beyond path compression) achieved by the combination of collapsing tree-like regions, splitting half-decision nodes, and converting non-decision nodes to edges. Percentage reduction is computed by (Old # edges - New # edges)/(Old # edges).

As expected, increasing read lengths leads to improved ability to reconstruct a genome: with reads of length 100 only 22 of the chromosomes have a unique solution, while 85 have a unique solution using reads of length 500, and over a quarter (120) have a unique solution with 1000 nt reads.

This is an encouraging result and demonstrates how, in the limit, increasing the read length will eventually resolve ambiguities in the genome. However, it also demonstrates the limitations of short-range paired-end protocols. If 1000 nt reads are not sufficient to fully disambiguate the repeat structure of most genomes, then short-range paired-end reads cannot either if the separation between the endpoints of the reads is less than 1000 nt. In fact, an assembly of short-range paired-end reads is likely to perform significantly worse than an assembly of long reads with corresponding length, since long reads are a conservative approximation of paired-end data. In particular, paired-end reads have additional sources of uncertainty (variation in the separation between the reads, chimeric pairs, etc) and substantial algorithmic challenges (efficiently finding unique paths of consistent separation, accurately resolving short tandem repeats, etc). Nevertheless, even a conservative approximation is sufficient to maintain our results as a theoretically strong upper bound on the potential for assembly.

### Number of reconstructions consistent with genome graphs

The number of strings that are consistent with a given de Bruijn graph is a reasonable estimation of the complexity of reassembling the genome given the information contained in perfect reads of a particular length. The more strings that are consistent with the graph, the more uncertain we are that a given string is the correct one. The short-read assembly problem can be completely solved, given only reads (and mate pairings), if and only if there is only a single string consistent with the de Bruijn graph.

The number of strings (*G*) consistent with a de Bruijn graph *G *is related, but not equal to, the number of unique Eulerian circuits. There may be many more Eulerian circuits than solution strings because the distinct orderings of *edges *that differentiate Eulerian circuits can lead to the same ordering of the *nodes*, and thus the same DNA sequence. Parallel edges are more than a theoretical issue: across the 2, 856 graphs considered (constructed using various read lengths), on average 18% of the edges had multiplicities ≥ 2. Therefore, it is important to correct for the common case of parallel edges.

**Figure 2:**
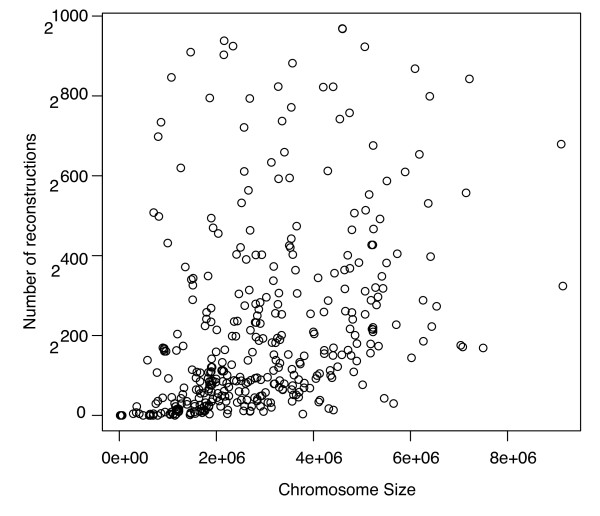
**Number of words consistent with genome graphs**. The size of the solution space for each chromosome using reads of length 50 nt. Only the 365 chromosomes that had fewer than 2^900 ^possible reconstructions are shown.

In Figure 2, we plot (*G*) for the de Bruijn graphs *G *constructed from length-50 reads. The number of possible reconstructions is often astronomical. Only the 365 chromosomes with fewer than 2^900 ^possible reconstructions are shown. As one would expect, the longer genomes generally have more solution words. However, this is not always the case. For example, *Yersinia pestis *appears much more complicated to reconstruct than *Escherichia coli*, even though both genomes are about the same size (4.4 Mb). Hence, one cannot use genome size as the sole indicator of assembly complexity. In fact, the genome of *Y. pestis *contains a large number of insertion sequences [34], and these insertion sequences complicate its de Bruijn graph.

### Achievable N50 contig size

Repeats cause ambiguities in the ordering of genome segments. The location and distribution of the repeats affects the quality of the assembly: many repeats localized to a small region (as in CRISPR [35] elements, for example) make that region difficult to assemble correctly, but do not disrupt the global organization of the genome. Conversely, repeats spread throughout the genome divide the sequence into many, smaller blocks whose order cannot be determined.

To determine what fraction of a typical genome can be assembled without error using reads of a given length, we extract from a simplified genome graph a set of contiguous sequences (*contigs*) that can be unambiguously assembled as discussed in the Methods section. From this set of idealized contigs, we compute the N50 size, a standard measure used to assess the quality of genome assemblies. Here, to facilitate comparison across hundreds of genomes, we divide the N50 score by the length of the chromosome to get the N50 contig size as a fraction of the total genome length. We we refer to this slightly modified measure as the *relative *N50.

**Figure 3:**
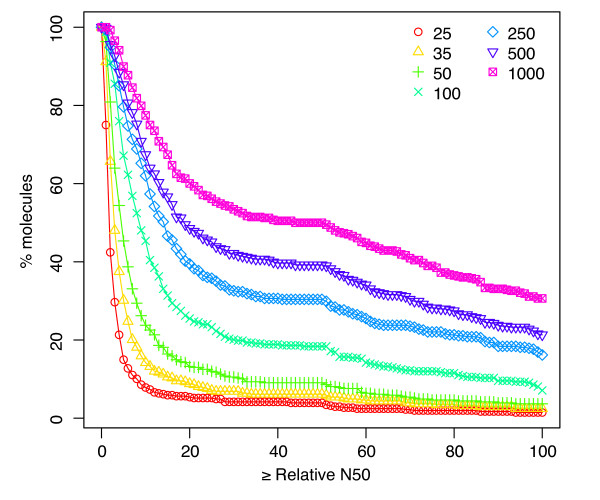
**Relative N50 size**. Cumulative histogram plotting the relative N50 size (x-axis; see text for definition) against the percentage of chromosomes (y-axis) for which the contigs achieve an N50 size at least that large. For example, approximately 40% of chromosomes yield a relative N50 contig size of at least 50% of the genome length when using 500-nt reads.

As expected, longer reads lead to a larger number of long contigs, and a corresponding increase in N50 size. Figure 3 plots the fraction of chromosomes for which the relative N50 was at least a given value. With 500 or 1000 nt long reads, 25-35% of the chromosomes can be nearly completely reconstructed in the absence of error. For 1000-nt reads, the median relative N50 size is 47% of the genome length, indicating that long contigs are often achievable. With 25-nt reads, however, the median relative N50 size is only 1.14% of the genome length. Despite this low N50 score, we will see in the next section that one can still reconstruct entire genes from even 25-nt data. Median relative N50 sizes for other read lengths are given in Table 2. These idealized N50 sizes are a practical upper bound on what can be achieved with reads of varying sizes and provide a benchmark against which to compare the success of assembly methods.

**Table 2:** Median N50 and reconstructible genes.

*k*	N50 (%)	Genes (%)
25	1.14	96.29
35	2.41	98.12
50	3.90	98.94
100	8.12	99.51
250	13.52	99.84
500	18.03	100
1000	46.57	100

Median N50 as a percentage of the chromosome size and median number of genes that are reconstructible for various read lengths *k*.

### Possibility of gene reconstruction

**Figure 4:**
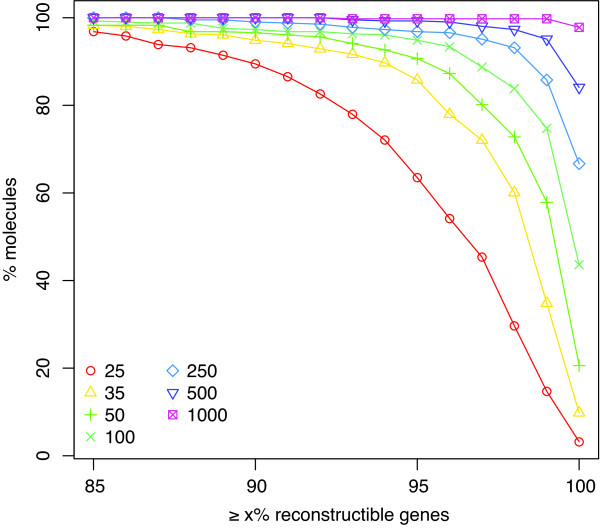
**Fraction of reconstructible genes**. Cumulative histogram plotting a percentage of genes (x-axis) against the percentage of chromosomes for which at least that many genes can be reconstructed. When *k *= 1000, nearly all chromosomes have all their genes completely reconstructed. When *k *= 25, the number of reconstructible genes falls off quicker, but many genes can still be reconstructed: 90% or more of the genes can be reconstructed in 89% of the chromosomes when *k *= 25.

Surprisingly, most genes in most of the genomes are reconstructible, as defined above, even when using very short reads. Figure 4 plots, for various read lengths, the percentage of chromosomes for which at least a given number of genes are reconstructible. Even with reads as short as 25 bases, at least 85% of all genes are reconstructible in almost all chromosomes. The biggest incremental improvement in reconstructibility comes from increasing read lengths from 25 nt to 35 nt. If 100 nt reads are used, 98.7% of genes are reconstructible in the average chromosome. The median number of reconstructible genes is shown in Table 2 for various read lengths. This successful gene reconstruction is further evidence that *de novo *short-read sequencing can yield useful information, even with very short read lengths.

A very large fraction of the genes that are not reconstructible by this definition were made non-reconstructible by mobile genetic elements. The non-reconstructible genes were statistically enriched for annotations related to transposases, insertion sequences (IS), phages, and integrases for read lengths 25, 35, 50, 100, 250, 500, and 1000 (all P-values < 10^-50^, hypergeometric distribution). For example, at 100-nt reads across all genomes, 49% of the non-hypothetical, non-reconstructible genes have the phrase "transpos" within their description (in the GenBank .ptt file). An additional 18% are tagged as insertion sequences. Thus, at least 67% of the difficulty in reconstructing the protein-coding genes using 100-nt reads stems from such mobile genetic elements. For other read lengths, the percentage of the non-reconstructible genes tagged as transposase- or IS-related ranges from 25% (25-nt reads) to 77% (500-nt reads). Conversely, most transposase genes are not reconstructible using reads *= *50-nt long: at 50-nt reads, 51% of transposase genes and 72% of IS-related genes were non-reconstructible.

The remaining non-reconstructible genes may be of interest on their own. Repeats within and nearby genes are one of the mechanisms through which prokaryotes achieve phase variation [36], for example, in order to evade the immune system. In addition, this idealized assessment of the ability to assemble coding regions gives us another benchmark against which to compare assemblies constructed from noisy data. Given these results, we should expect assemblies to contain the complete sequences of a high fraction of protein-coding genes.

## Conclusions

We present a comprehensive examination of the difficulty of reconstructing prokaryotic genomes from short substrings based on several measures derived from an idealized analysis of the genome reconstruction problem. The resulting simplified genome graphs for 375 organisms are publicly available at http://www.cbcb.umd.edu/research/complexity/. These graphs may be a starting point for additional repeat analysis [26].

These graphs provide a strong theoretical upper bound on the performance of short read assembly, and therefore provide a yardstick against which to measure the performance of current and future genome assemblers. In particular, while computational methods and laboratory protocols for minimizing sequencing errors and uneven coverage are improving, our results reveal the unavoidable fundamental limitations of assembly from short reads. Even paired-end reads, which have been extremely useful for resolving certain classes of repeats, will not alone solve the assembly problem for most organisms.

Finally our analyses show that even though there may be an astronomical number of consistent genome reconstructions assembled from short reads, even extremely short reads (25 bp) are sufficient to correctly reconstruct the majority of genes in an organism under idealized conditions. For many genome studies, this may be sufficient to satisfy the goals of the project, but caution is prudent if computing a full unambiguous genome reconstruction from short reads is required. If practical results are not as impressive, those results can be attributed to either non-ideal data or deficiencies in the algorithms, rather than inherent limitations. By better understanding these limitations with idealized data, we can also begin to investigate what additional information (such as optical maps [37]) is most profitably added to short-read sequencing data to make these new technologies as useful as possible.

## Authors' contributions

CK, MCS, and MP designed the study and wrote the manuscript. CK and MCS performed the computational experiments. All authors read and approved the final manuscript.
